# Genome analysis of the candidate phylum MBNT15 bacterium from a boreal peatland predicted its respiratory versatility and dissimilatory iron metabolism

**DOI:** 10.3389/fmicb.2022.951761

**Published:** 2022-08-04

**Authors:** Shahjahon Begmatov, Alexey V. Beletsky, Svetlana N. Dedysh, Andrey V. Mardanov, Nikolai V. Ravin

**Affiliations:** ^1^Institute of Bioengineering, Research Center of Biotechnology of the Russian Academy of Sciences, Moscow, Russia; ^2^Winogradsky Institute of Microbiology, Research Center of Biotechnology of the Russian Academy of Sciences, Moscow, Russia

**Keywords:** metagenome, candidate phylum, MBNT15, genome taxonomy, iron reduction, peatland

## Abstract

Uncultured bacteria of the candidate phylum MBNT15, distantly related to *Desulfobacterota*, have been identified in a broad range of mostly organic-rich aquatic environments. We assembled a near-complete genome of a member of MBNT15 from a boreal peatland metagenome and used genomic data to analyze the metabolic pathways of this bacterium and its ecological role. This bacterium, designated SHF-111, was predicted to be rod shaped, it lacks flagellar machinery but twitching motility is encoded. Genome-based phylogenetic analysis supported the phylum-level classification of the MBNT15 lineage. Genome annotation and metabolic reconstruction revealed the presence of the Embden–Meyerhof, Entner–Doudoroff and pentose phosphate pathways, as well as the complete tricarboxylic acid (TCA) cycle, and suggested a facultatively anaerobic chemoheterotrophic lifestyle with the ability to ferment peptides, amino acids, fatty acids and simple sugars, and completely oxidize these substrates through aerobic and anaerobic respiration. The SHF-111 genome encodes multiple multiheme c-type cytochromes that probably enable dissimilatory iron reduction. Consistently, the relative abundance of MBNT15 in peatlands positively correlated with iron concentration. Apparently, in the wetland ecosystem, MBNT15 representatives play the role of scavengers, carrying out the complete mineralization of low molecular weight organic substances formed as a result of microbial degradation of complex polymeric substrates. Comparative genome analysis of the MBNT15 phylum revealed that vast majority of its members are capable of aerobic respiration and dissimilatory iron reduction and some species also can reduce sulfur and nitrogen compounds, but not sulfate. Based on phylogenetic and genomic analyses, the novel bacterium is proposed to be classified as *Candidatus* Deferrimicrobium borealis, within a candidate phylum Deferrimicrobiota.

## Introduction

Culture-independent surveys of microbial communities based on sequencing of 16S rRNA gene have been widely utilized to characterize the global patterns of microbial diversity in nature and revolutionized our understanding of diversity of microbial world (Hugenholtz et al., 1998; Parks et al., 2017; Thompson et al., 2017). These studies revealed that the great majority of microorganisms are uncultured; many major microbial lineages still lack cultivated representatives and are usually poorly characterized (Hugenholtz et al., 1998; Dick and Baker, 2013; Eloe-Fadrosh et al., 2016; Solden et al., 2016; Parks et al., 2017). However, these uncultured lineages represent a valuable source of information about the evolution of microbial world, unknown metabolic pathways and ecological functions in natural ecosystems (Rinke et al., 2013; Hu et al., 2016; Solden et al., 2016; Mukherjee et al., 2017).

Understanding of ecophysiology of uncultured microorganisms has been progressed with the developments in next-generation sequencing and advent of novel omics approaches such as sequencing-based metagenomics, single-cell genomics, metatranscriptomics, etc. (Sheik et al., 2014; Moitinho-Silva et al., 2017; Jochum et al., 2018). New approaches resulted in expansion of the amount of known bacterial and archaeal genomes from a variety of environments, provided an invaluable resource for building a genome-based taxonomic system and contributed to the understanding of the metabolic potential and ecological functions of uncultivated microorganisms (Wrighton et al., 2012; Rinke et al., 2013; Brown et al., 2015; Anantharaman et al., 2016; Hug et al., 2016; Parks et al., 2017, 2018; Nayfach et al., 2021).

The candidate bacterial phylum MBNT15 was originally revealed by the phylogenetic analysis of 16S rRNA gene sequences obtained from a rice paddy soil in 2009 (GenBank FJ538146). Over the next 12 years 16S rRNA gene sequences assigned to this division have been detected by molecular methods in various natural environments, including soils, rhizosphere, peatlands, marine, and freshwater environments and sediments. At present, the SILVA database (v. 138.1) contains 4,620 16S rRNA gene sequences assigned to MBNT15. The environments colonized by MBNT15 bacteria are highly diverse, but usually they are rich in partly decomposed organic matter and vary in oxygen concentration. To the best of our knowledge, none of the studies revealed the MBNT15 lineage as a dominant member of microbial communities.

MBNT15 is currently recognized as a phylum-level lineage in both the 16S rRNA gene-based SILVA database and the genome-based [Genome Taxonomy Database (GTDB)] taxonomy (Quast et al., 2013; Parks et al., 2018). The GTDB system defines this lineage as the only class MBNT15 in the candidate phylum designated Desulfobacterota_E (release R202). Such definition reflects the close phylogenetic relationships between MBNT15 and *Deltaproteobacteria*. In a recently proposed taxonomic reclassification of the class *Deltaproteobacteria* into four novel phyla, *Desulfobacterota, Myxococcota, Bdellovibrionota*, and SAR324, the MBNT15 lineage, as well as its closest relative, UBP10 (Parks et al., 2017) (known as GR-WP33-30 in the 16S rRNA phylogenies), are considered as classes within the phylum *Desulfobacterota* (Waite et al., 2020). However, the UBP10 division has been classified as a candidate phylum *Binatota* (Chuvochina et al., 2019), and both MBNT15 and *Binatota* are currently recognized as phylum-level divisions in the GTDB.

Although MBNT15 bacteria remain uncultured, some information about their biology was obtained from metagenomics studies. At present, 32 genomes of MBNT15 bacteria are available in GTDB, but all of them are incomplete and represented by dozens-hundreds of contigs. The first data on the genetic potential of this candidate phylum were obtained from metagenomic analysis of sediments from Middle Park Beach, Port Philip Bay, Australia (Chen et al., 2021). MBNT15 was generally rare in the sediments (<1% of 16S rRNA gene reads) and only thrived in deeper anoxic layers, indicating anaerobic lifestyle. Two metagenome-assembled genomes (MAGs), both assembled as medium quality drafts, were assigned to the MBNT15 lineage. Genome annotations based on homology-based searches and analysis using METABOLIC v.4.0 tool indicated that MBNT15 bacteria are obligate anaerobes that couple H_2_ and acetate oxidation to nitrate reduction (Chen et al., 2021). Both genomes also contained incomplete set of genes of the Wood–Ljungdahl pathway for carbon fixation. MBNT15 appeared to be habitat specialists that thrive in anoxic deep sediments, but lack the metabolic capabilities to proliferate in transiently oxygenated surface sediments (Chen et al., 2021). Very little is known about the functional properties of MBNT15 in other ecosystems. To the best of our knowledge, other physiological information about this candidate phylum is currently not available and no other draft genomes were used for the metabolic reconstruction of MBNT15 bacteria.

Although the range of habitats colonized by MBNT15 is not yet fully understood, these bacteria are commonly found in peat accumulating wetlands (peatlands) in 16S rRNA gene profiling studies. Wetlands cover about 5–8% of the Earth’s land surface (Mitsch et al., 2013) and provide important ecosystem services such as wildlife habitat, water purification, and flood control (He et al., 2015). Peatlands cover about 4 million km^2^ worldwide, mostly in temperate-cold climates in the northern hemisphere, particularly in Russia, Canada, and the United States (Limpens et al., 2008). Peatlands are classified into various types based on vegetation and trophic status. The two contrasting types of peatlands are raised bogs, fed solely by precipitation, and fens, which are mainly filled with groundwater and runoff (Aselmann and Crutzen, 1989; Dedysh et al., 2006). Raised bogs are highly acidic (pH around 4), poor in nutrients and dominated by *Sphagnum* mosses. Eutrophic fens are generally less acidic and more nutrient-rich than bogs, with sedges and grasses being the main vegetation type.

Russia is among the countries with the largest area of mires in the world. In the North European part of the country their area is about 15 million hectares (Sirin et al., 2017). Many large mire regions include closely located peatlands of various types, mainly raised bogs and eutrophic fens.

In our previous studies, we characterized microbial communities in several peatlands in European North Russia using 16S rRNA gene profiling. It was found that microbiomes of peat samples from raised bogs were highly similar to each other and clearly differed from those in eutrophic fens. *Acidobacteriota*, mostly members of the orders *Acidobacteriales* and *Bryobacterales*, dominated in raised bogs, while *Anaerolineae* (*Chloroflexi*), *Vicinamibacteria*-, and *Blastocatellia*-affliated *Acidobacteriota*, *Rokubacteria*, uncultivated group OM190 of the *Planctomycetota* and several groups of *Proteobacteria* were typical for the fens (Ivanova et al., 2020). Members of the MBNT15 lineage were also found in microbial communities of some peat samples (Ivanova et al., 2020; Rakitin et al., 2022).

In this study, we used metagenomic sequencing to assemble a near-complete genome of bacterium of this candidate phylum. Genome data were used for reconstruction of the metabolic pathways of the MBNT15 bacterium, comparative genome analysis of this uncultured bacterial lineage and prediction of its ecological roles.

## Materials and methods

### Detection of MBNT15 in ten peatlands of European North Russia

Microbial diversity patterns in two types of boreal peatlands, raised bogs and eutrophic fens, analyzed using the 16S rRNA gene profiling, were described previously (Dedysh et al., 2021) and deposited in sequence read archive (SRA) under the accession numbers SRR11280489–SRR11280524 (BioProject PRJNA610704). The peat samples for 16S rRNA gene profiling were obtained from four raised bogs (Shichengskoe, Piyavochnoe, Barskoe, and Alekseevskoe) and six eutrophic fens (Shichengskoe, Piyavochnoe, Rodionskoe, Ileksa, Povreka, and Charozerskoe) located in the Vologda region of European North Russia, within the zone of middle taiga. Detail description of the sampling sites was reported previously (Ivanova et al., 2020; Dedysh et al., 2021), as well as their physicochemical characteristics (Dedysh et al., 2021).

The aforementioned 16S rRNA gene sequences were clustered into operational taxonomic units (OTUs) at 97% identity using the USEARCH v. 11 program (Edgar, 2010). Low-quality reads, chimeric sequences, and singletons were removed by the USEARCH algorithm. To calculate OTU abundances, all reads obtained for a given sample (including singletons and low-quality reads) were mapped to OTU sequences at a 97% global identity threshold by USEARCH. The taxonomic assignment of OTUs was performed by searching against the SILVA v.138 rRNA sequence database using the VSEARCH v. 2.14.1 algorithm (Rognes et al., 2016).

### Correlations between abundance of MBNT15 and chemical properties of peat

MicrobiomeSeq v. 0.1^1^ R package was used to calculate Pearson and Spearman correlation coefficients between abundances of MBNT15 OTUs and environmental factors. The significance of the correlation was tested by calculating the *p*-values, adjusted for multiple comparisons using Benjamin and Hochberg method in MicrobiomeSeq v. 0.1. Correlations were considered significant if they had an adjusted *p*-value <0.05.

### Sequencing of metagenomic DNA and assembly of metagenome-assembled genomes

Total genomic DNA was isolated from 250 mg of peat sample using DNeasy PowerSoil Kit (Qiagen, Hilden, Germany) and sequenced using the Illumina HiSeq2500 platform according to the manufacturer’s instructions (Illumina Inc., San Diego, CA, United States). The sequencing of a paired-end (2 × 150 bp) TruSeq DNA library generated 390,190,181 read pairs (∼117.26 Gb). Adapter removal and trimming of low-quality sequences (*Q* <30) were performed using Cutadapt v.3.4 (Martin, 2011) and Sickle v.1.33,^2^ respectively.

In addition, genomic DNA was sequenced on a MinION device (Oxford Nanopore, Oxford, United Kingdom) using the ligation sequencing kit 1D and FLOMIN110 cells. Sequencing resulted in 4,549,065 reads with a total length of ∼16 Gbp. Nanopore reads were *de novo* assembled using Wtdbg2 v.2.5 (Ruan and Li, 2020). The sequences of obtained contigs were polished using Illumina reads with two iterations of NextPolish v.1.4.0 (Hu et al., 2020).

The obtained contigs were binned into MAGs using MetaBAT v. 2.15 (Kang et al., 2019). The assembled MAGs were taxonomically classified using the Genome Taxonomy Database Toolkit (GTDB-Tk) v.1.5.0 (Chaumeil et al., 2019) and GTDB (Parks et al., 2022). CheckM 1.1.3 (Parks et al., 2015) was used to check completeness and contamination values of obtained MAGs.

### Annotation and analysis of genomes

Gene search and annotation of MAG were performed using the RAST server 2.0 (Brettin et al., 2015), followed by manual correction of the annotation by comparing the predicted protein sequences with the National Center for Biotechnology Information (NCBI) databases. The N-terminal signal peptides were predicted by Signal P v.5.0, and the presence of transmembrane helices was predicted by TMHMMv.2.0.^3^ The carbohydrate active enzymes and transporters were identified using dbCAN (Yin et al., 2012) and transporter classification database (Saier et al., 2021), respectively. The HydDB web tool was used for hydrogenase classification and analysis (Søndergaard et al., 2016). The presence of particular metabolic pathways in MAGs was analyzed using the Distilled and Refined Annotation of Metabolism (DRAM) tool (Shaffer et al., 2020).

The average nucleotide identity (ANI) between the selected genomes was calculated using appropriate script from the Enveomics Collection (Rodriguez-R and Konstantinidis, 2016).

For genome-based phylogenetic analysis the GTDB-Tk v.1.5.0 tool was used to find single-copy marker genes in the MAGs and to construct a multiple sequence alignment of concatenated single-copy gene sequences, consisting of MBNT15 MAG obtained in this study and all species from the GTDB. A part of the multiple sequence alignment generated in GTDB-Tk was used to construct a phylogenetic tree with PhyML v.3.3 (Guindon et al., 2010) using default parameters. The level of support for internal branches was assessed using the Bayesian test in PhyML.

## Results and discussion

### MBNT15 abundance, diversity, and distribution in peatlands

Members of the MBNT15 were detected in all six studied fens but were absent from four raised bogs (Supplementary Table 1). This lineage was especially abundant in peat samples from Shichengskoe (up to 1.4% of total reads), Rodionskoe (up to 2.1%), and Charozerskoe fens (up to 1.2%), was less numerous in the Ileksa (up to 0.4%), and represented minor groups in the Povreka and Piyavochnoe fens (less than 0.1%).

Using a sequence identity of 97%, only two OTUs assigned to the MBNT15 were identified in the studied peat samples. One OTU was detected in all fens while the second OTU, displaying 96.58% sequence identity to the first one, was found only in peat samples from the Rodionskoe fen where it accounted for up to one third of all MBNT15 reads (Supplementary Table 1).

The SILVA database (v. 138.1) contains 4,620 16S rRNA gene sequences assigned to the candidate phylum MBNT15, and information about isolation source is available for 2,608 of them. The majority of these sequences were retrieved from soil and rhizosphere (27%), sediments (27%), and peatlands (10%).

### Correlations between the chemical characteristics of peat and the relative abundance of MBNT15

**FIGURE 1:**
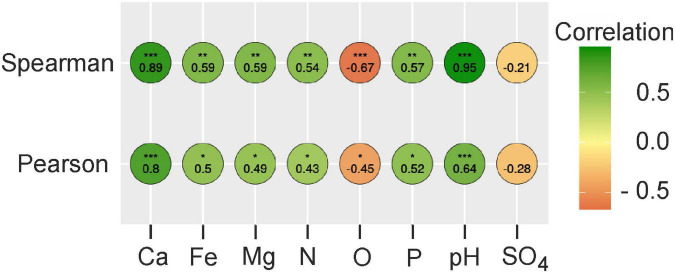
The correlation matrix based on Spearman’s and Pearson’s correlation analysis between peat chemistry and relative abundances of MBNT15. The color of circles represents the correlation strength. Significant correlations are indicated by asterisks; adjusted p-value confidence level * < 0.05; ** < 0.01; *** < 0.001. N, total nitrogen; O, organics; P, phosphorus.

To uncover the possible influence of peat chemical characteristics on the abundance of MBNT15, we performed a correlation analysis with a further significance test. Both the Pearson and Spearman analysis revealed a positive correlation between the relative abundance of MBNT15 with total nitrogen, pH and the concentrations of Fe, Ca, Mg, and phosphorous. A negative correlation was observed with the total organic carbon (Figure 1).

### Reconstruction of the complete genome of a member of the candidate phylum MBNT15

To obtain MAGs of microbial community members, we sequenced the metagenome of a peat sample from the Shichengskoe fen using the techniques of Illumina and Oxford Nanopore. According to the 16S rRNA gene profiling, the microbial community of this sample comprised a single OTU assigned to MBNT15 with a total share of 1.6%. Analysis of the taxonomic affiliation of the obtained MAGs showed that one of them, designated SHF-111, belongs to the MBNT15 lineage. This MAG consisted of six contigs with a total length of 2,677,482 bp and was sequenced to 182-fold average coverage. The relative abundance of this genotype in the community, defined as a fraction of this MAG in the metagenome, was about 0.53%. CheckM estimated that the completeness of this MAG was 100%, with a possible 1.68% contamination. Thus, the SHF-111 genome met the recently proposed criteria (Bowers et al., 2017) for a “high-quality” MAG. This is the best assembly of MBNT15 genomes available in GTDB R202 (Supplementary Table 2).

A single 23S-5S ribosomal RNA operon, a single distantly located 16S rRNA gene, and 39 transfer RNA (tRNA) genes coding for all of the 20 amino acids were identified. Genome sequence annotation predicted 2,479 protein-coding genes. Only five genes encoded mobile element-related proteins and the genome lacked a clustered regularly interspaced short palindromic repeats (CRISPR) system.

The genome of the SHF-111 bacterium lacked the genes encoding the flagellar machinery and chemotaxis. However, a set of genes necessary for the generation of type IV pili have been identified. Such pili enable twitching motility and attachment of cells to solid surfaces, including insoluble growth substrates (Mandlik et al., 2008). SHF-111 cells are predicted to be rod shaped, based on the identification of genes encoding the rod shape-determining proteins MreBCD, and RodA.

### Phylogenetic placement of the SHF-111 bacterium

A search against GTDB placed the SHF-111 MAG in the candidate phylum Desulfobaterota_E, class, order, and family designated MBNT15, and finally in the genus CG2-30-66-27. The closest relative of SHF-111 with an ANI of 90.6% is Deltaproteobacteria bacterium MAG S05.Bin005 (GCA_011391555.1), obtained from the lacustrine sediment metagenome in Tibet, China. ANI values between SHF-111 and other genomes assigned to g_CG2-30-66-27 ranged from 85.3 to 90.7% (Supplementary Table 3) and according to the criteria proposed by Konstantinidis et al. (2017) for delineation of the phylogenetic position of uncultivated microorganisms SHF-111 represented a novel species in this genus.

**FIGURE 2:**
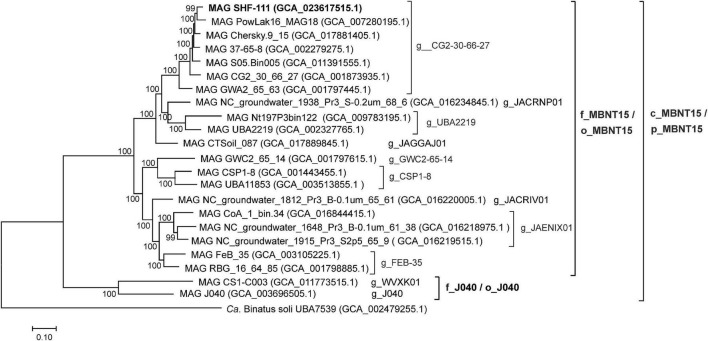
Genome-based phylogeny of the candidate phylum MBNT15. Taxonomy is shown according to the GTDB (p,_phylum; c_class; o_order; f_,family; and g_,genus). The genome sequence of “*Candidatus* Binatus soli”, a member of the sister candidate phylum Binatota (UBP10), was used to root the tree.

To analyze the phylogeny of the candidate phylum MBNT15, we constructed a phylogenetic tree based on the concatenated sequences of 120 conserved marker genes, including the SHF-111 genome, six other species from g_CG2-30-66-27 and 15 other MBNT15 genomes, representing all species-level lineages recognized in GTDB (Figure 2). All genera, families and orders proposed by the GTDB within the MBNT15 were represented by distinct monophyletic branches.

### Central metabolic pathways

**FIGURE 3:**
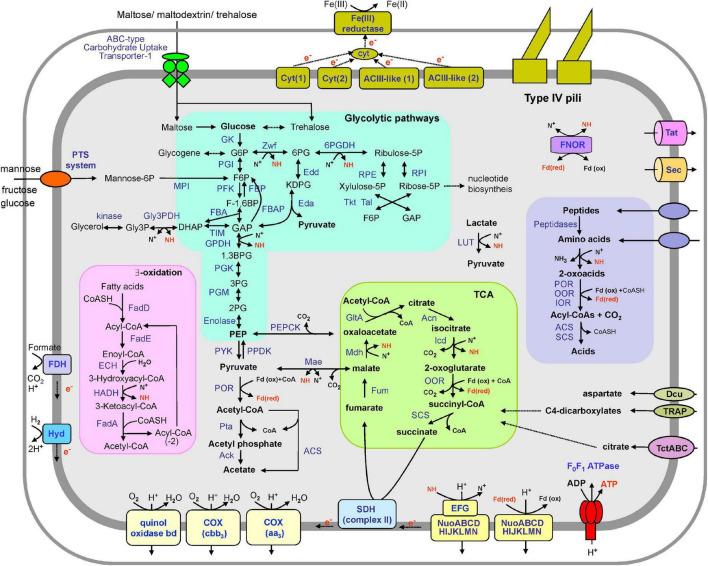
An overview of the metabolism of the SHF-111 bacterium. Enzymes and proteins identified in the genome are in blue, energy-rich intermediate compounds are in red. Enzyme abbreviations: GK, glucokinase; PGI, Glucose-6-phosphate isomerase; PFK, phosphofructokinase; FBA, fructose-bisphosphate aldolase; TIM, triose phosphate isomerase; GPDH, glyceraldehyde 3-phosphate dehydrogenase; PGK, phosphoglycerate kinase; PGM, phosphoglycerate mutase; PYK, pyruvate kinase; PPDK, pyruvate phosphate dikinase; FBP, fructose-1,6-bisphosphatase; FBAP, fructose-1,6-bisphosphate aldolase/phosphatase; Zwf, glucose-6-phosphate dehydrogenase; Edd, phosphogluconate dehydratase; Eda, 2-dehydro-3-deoxy-phosphogluconate aldolase; 6PGDH, 6-phosphogluconate dehydrogenase; RPE, ribulose-5-phosphate epimerase; RPI, ribulose-5-phosphate isomerase; Tal, transaldolase; Tkt, transketolase; POR, pyruvate ferredoxin oxidoreductase; Pta, phosphate acetyltransferase; Ack, acetate kinase; ACS, acetyl-CoA synthetase (ADP-forming); LUT, lactate dehydrogenase (YkgEFG-type); GltA, citrate synthase; Acn, aconitase; Icd, isocitrate dehydrogenase; OOR, 2-oxoglutarate ferredoxin oxidoreductase; SCS, succinyl-CoA ligase; SDH, succinate dehydrogenase; Fum, fumarate hydratase; Mdh, malate dehydrogenase; Mae, malic enzyme; PEPCK, phosphoenolpyruvate carboxykinase; COX, cytochrome c oxidase; ACIII-like (1) and ACIII-like (2), two membrane-linked complexes similar to alternative complex III; Hyd, [NiFe] hydrogenase; FDH, formate dehydrogenase; FadD, fatty acid-CoA ligase; FadE, acyl-CoA dehydrogenase; ECH, enoyl-CoA hydratase; HADH, 3-hydroxyacyl-CoA dehydrogenase; FadA, 3-ketoacyl-CoA thiolase; FNOR, ferredoxin NADP(H) oxidoreductase; IOR, indolepyruvate ferredoxin oxidoreductase; Cyt(1) and Cyt(2), two membrane-linked complexes comprising multiheme c-type cytochromes. Other abbreviations: G6P, glucose-6-phosphate; F6P, fructose-6-phosphate; F-1,6BP, fructose 1,6-bisphosphate; GAP, glyceraldehyde-3-phosphate; 1,3BPG, 1,3-bisphosphoglycerate; 3PG, 3-phosphoglycerate; 2PG, 2-phosphoglycerate; PEP, phosphoenolpyruvate; 6PG, 6-phosphogluconate; KDPG, 2-keto-3-deoxy-6-phosphogluconate; Fdox/Fdred, ferredoxin, oxidized and reduced form; N^+^, NAD(P)^+^; NH, NAD(P)H; Cyt, cytochrome; CoA, coenzyme A.

To uncover the potential functions of the SHF-111 bacterium, we constructed a metabolic model based on the annotated genome (Figure 3 and Supplementary Table 4). The SHF-111 genome contains a complete set of genes encoding enzymes of an Embden–Meyerhof glycolytic pathway and gluconeogenesis. It should be noted that some enzymes of the glycolysis and gluconeogenesis pathways (e.g., glucose-6-phosphate isomerase, pyruvate kinase, phosphoenolpyruvate synthase, and fructose-1,6-bisphosphatase) are represented by several genes. The Entner–Doudoroff glycolytic pathway was also present, including genes for glucose-6-phosphate 1-dehydrogenase, 6-phosphogluconolactonase, phosphogluconate dehydratase, and 2-dehydro-3-deoxy-phosphogluconate aldolase. Moreover, the pentose phosphate pathway including both oxidative and non-oxidative branches was also encoded.

Glycogen may be used as a storage polymer as suggested by the presence of genes for key enzymes involved its synthesis, including glycogen synthase, and 1,4-alpha-glucan (glycogen) branching enzyme. Enzymes of two pathways for the synthesis of another storage polysaccharide, trehalose, were encoded: OtsAB (trehalose-6-phosphate synthase and trehalose-6-phosphate phosphatase), as well as TreYZ (malto-oligosyltrehalose synthase and glycosyltrehalose trehalohydrolase). Therefore, these polymers may serve as carbon and energy storage, which can be utilized to mitigate fluctuations in substrate availability (Argüelles, 2000; Wilson et al., 2010).

Pyruvate could be decarboxylated to acetyl coenzyme A (CoA) by pyruvate:ferredoxin oxidoreductase. Acetyl-CoA could be converted to acetate with the concomitant production of ATP either through a one-step reaction with acetyl-CoA synthetase (ADP-forming) or through a two-step reaction involving phosphate acetyltransferase and acetate kinase.

Complete oxidation of organic substrates could be enabled by the tricarboxylic acid (TCA) cycle, all genes of which were identified in the SHF-111 genome. Two additional genes coding for enzymes linking the TCA and glycolysis were identified, namely the malic enzyme that decarboxylate malate to pyruvate and the phosphoenolpyruvate carboxykinase, that converts oxaloacetate into phosphoenolpyruvate and carbon dioxide. The cycle probably operates only in the oxidative direction as evidenced by the absence of citrate lyase.

Autotrophic CO_2_ fixation could be performed through the Wood–Ljungdahl pathway (Ragsdale and Pierce, 2008). Although some of the relevant genes were identified in the SHF-111 genome, the lack of formate-tetrahydrofolate ligase and complete CO dehydrogenase/acetyl-CoA synthase complex indicates the absence of this pathway. Analysis of the genomes of other members of the phylum MBNT15 also showed the absence of complete Wood–Ljungdahl pathway. The key enzymes of the Calvin–Benson–Bassham and 3-hydroxypropionate pathways were not identified.

### Respiratory electron transfer chains

The major components of the electron transfer chain for energy generation via oxidative phosphorylation are encoded in the SHF-111 genome, namely the proton-translocating NADH:quinone oxidoreductase complexes, membrane-bound succinate dehydrogenase and terminal oxidases. The transmembrane ion gradient generated by this chain may be used for ATP synthesis by an F_0_F_1_-type ATPase. The SHF-111 genome contains two main clusters encoding most of the subunits of NADH dehydrogenase, *nuoABCDEFGHIJKLMN* and *nuoABCDHIJKLMN*. Although these two clusters follow each other in the genome, the similarity of the amino acid sequences of the corresponding subunits ranges only from 31 to 60%, indicating that the two clusters are not the result of a recent duplication. The absence of genes encoding the NuoEFG subunits which are known to form the NADH-interacting module (Sazanov, 2007) in the second cluster suggest the possibility that it encodes an alternative membrane-bound proton-transporting complex accepting electrons from a donor other than NADH (e.g., reduced ferredoxin).

The terminal oxygen reductases in the SHF-111 bacterium are represented by two proton-translocating cytochrome c oxidases and a quinol oxidase *bd* complex. Two cytochrome c oxidases belong to the *aa3* and *cbb3* types. These oxidases differ in their affinity to oxygen: while *aa3*-type enzymes have a low affinity and are usually used by aerobic microorganisms, *cbb3*-type oxidases usually have a very high affinity for oxygen, enabling respiration under microaerobic conditions (Pitcher et al., 2002). The predicted ability of the SHF-111 bacterium to grow under aerobic conditions is consistent with the presence of superoxide dismutase and catalase participating in protection against reactive oxygen species in aerobes.

Besides NAD(P)H and reduced ferredoxin, the oxidation of hydrogen and formate could supply electrons to the electron transport chain. The SHF-111 genome comprises genes encoding a membrane-linked [NiFe] group 1b hydrogenase that enables respiratory H_2_ uptake (Greening et al., 2016). Such hydrogenases are oxygen-sensitive; they are widespread in *Deltaproteobacteria* inhabiting anoxic soils, sediments, and marine waters (Greening et al., 2016). The oxidation of formate could be enabled by two formate dehydrogenases, comprising membrane-linking subunits.

### Possible growth substrates of SHF-111 bacterium

The search for potentially secreted glycosyl hydrolases that could enable the utilization of polysaccharides revealed the absence of enzymes containing N-terminal secretion signal peptides. A few encoded intracellular glycoside hydrolases (e.g., alpha amylase, amylomaltase, etc.) are likely involved in the synthesis and degradation of storage polysaccharides, glycogen, and trehalose. However, two sugar transport systems were identified in the SHF-111 genome. Sugar ABC transporter of the Carbohydrate Uptake Transporter-1 (CUT1) Family (TC: 3.A.1.1) could enable uptake of maltose, trehalose, and maltooligosaccharides (Bordignon et al., 2010). The phosphotransferase system of mannose family (TC: 4.A.6) could catalyze the phosphorylation of incoming sugar substrates such as glucose, mannose, and fructose, conjunction with translocation across the cell membrane (Rephaeli and Saier, 1980). Mannose-6-phosphate isomerase then converts mannose-6-phosphate to fructose 6-phosphate entering glycolytic pathways. The presence of ribokinase and the Leloir pathway suggest that ribose and galactose could be utilized as well. Genome analysis also revealed the presence of glycerol kinase and glycerol-3-phosphate dehydrogenase that suggest the possibility of using glycerol as a substrate.

Although the SHF-111 genome encodes no signal peptide-containing proteases that could enable extracellular hydrolysis of proteinaceous substances, multiple transport systems for an uptake of amino acids and short peptides were identified. The most numerous were branched-chain amino acid uptake ABC transporters of the hydrophobic amino acid uptake transporter (HAAT) Family (TC 3.A.1.4), represented by 11 *livJFGHM* gene clusters (Basavanna et al., 2009). One of them is linked to an operon encoding branched-chain alpha-keto acid dehydrogenase. This enzyme complex catalyzes the oxidative decarboxylation of deaminated derivatives of branched alpha-keto acids. An uptake of oligo-, tri-, and dipeptides could be performed by two ABC transporters of the Peptide/Opine/Nickel Uptake Transporter Family (TC 3.A.1.5). The SHF-111 genome also contains genes for amino acid transporters of the Polar Amino Acid Uptake Transporter Family (TC 3.A.1.3) and the Amino Acid-Polyamine-Organocation Superfamily (TC 2.A.3). Imported amino acids can be deaminated and converted into 2-oxoacids. These intermediates are then further oxidized by the 2-oxoacids: ferredoxin oxidoreductases to make acetyl-CoA or succinyl-CoA, which can then be cleaved to yield acetate or succinate with ATP generation (Fukui et al., 2005). Glycine can alternatively be degraded via a glycine cleavage system.

Besides transporters enabling the uptake of sugars and amino acids, the SHF-111 bacterium has multiple transport systems enabling utilization of di- and tricarboxylates of the TCA cycle. Genome analysis revealed the presence of the TRAP-type C4-dicarboxylate transport system (TC: 2.A.56). Such transporters catalyze the symport of C4-dicarboxylates (succinate, fumarate, and malate) and aspartate with H^+^ or Na^+^ (Janausch et al., 2002). Four gene clusters encodes transporters of the Tripartite tricarboxylate transporter TctABC (TTT) family (TC: 2.A.80). The prototype for this family, citrate transporter, was first described by Sweet et al. (1979) in the *Salmonella typhimurium*, and citrate uptake is the most commonly identified role of the other characterized TTT systems (Rosa et al., 2018). Transporter of the C4-Dicarboxylate Uptake (Dcu) Family (TC: 2.A.13) could transport aspartate, malate, fumarate and succinate and function as antiporters with any two of these substrates. Since this transporter is encoded in an operon with the gene for aspartase, its likely physiological function may be to catalyze aspartate: fumarate exchange during the utilization of aspartate as a nitrogen source (Six et al., 1994). Finally, the transport of organic di- and tricarboxylates of the TCA cycle as well as the dicarboxylate amino acid could be performed by a transporter of the Divalent Anion:Na^+^ Symporter (DASS) Family (TC 2.A.47).

Fatty acids can also serve as substrates for the growth of SHF-111 bacteria. This is consistent with the presence of a complete fatty acid beta-oxidation pathway, including multiple copies of genes for long-chain fatty acid-CoA ligase, acyl-CoA dehydrogenase, enoyl-CoA hydratase, 3-hydroxyacyl-CoA dehydrogenase and 3-ketoacyl-CoA thiolase. Fatty acids with an odd-number of carbons are oxidized in the same manner, but the final products are propionyl-CoA and acetyl CoA. The former could be carboxylated to methylmalonyl-CoA by propionyl-CoA carboxylase. Then methylmalonyl-CoA epimerase and methylmalonyl-CoA mutase convert it to succinyl-CoA entering the TCA cycle.

### Possible mechanisms of iron reduction

Genome analysis revealed no known reductases for anaerobic respiration with sulfate and other sulfur compounds, nitrate, nitrite and arsenate. However, the SHF-111 genome contains 32 genes encoding multiheme c-type cytochrome with up to 16 heme-binding motifs (Supplementary Table 5). Twenty-two of these cytochromes carry N-terminal signal peptides, indicating their export from the cytoplasm. Such c-type cytochromes play a key role in the electron transfer out of the cell to the extracellular electron acceptor in the well-studied dissimilatory Fe(III) reducing Gram-negative bacteria *Shewanella* and *Geobacter* (Shi et al., 2007; Richter et al., 2012). Six multiheme cytochromes contain up to three Ser/Thr-Pro-Ser/Thr motifs enabling binding to hematite, the mineral form of iron(III) oxide (Fe_2_O_3_), as described previously for an iron oxide reductase of *S. oneidensis* (Lower et al., 2008). Interaction with insoluble Fe(III) minerals could be also facilitated by the type IV pili, encoded by the SHF-111 genome.

**FIGURE 4:**
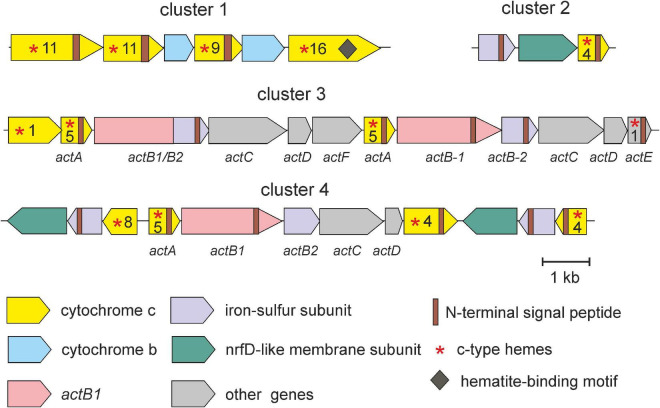
Clusters of genes possible involved in dissimilatory iron reduction in the SHF-111 bacterium. Genes are represented by arrows. The number of heme-binding motifs (CxxCH) is indicated after the asterisk.

Further genome analysis revealed several gene clusters that encode putative complexes involved in Fe(III) reduction (Figure 4). The first locus (cluster 1) comprises four genes encoding multiheme c-type cytochrome containing from 9 to 16 heme-binding motifs and two genes for cytochromes b, each containing four transmembrane domains. Three of c-type cytochrome contains N-terminal signal peptides and transmembrane helices indicating their export from the cytoplasm and membrane localization. Such complexes could accept electrons from the quinone pool and transfer them to periplasmic electron shuttles (Rothery et al., 2008). Genome analysis revealed several genes possibly encoding such periplasmic cytochromes c, containing N-terminal secretion signals but lacking membrane-anchoring helices. The fourth multiheme cytochrome in addition to 16 hemes contains three Ser/Thr-Pro-Ser/Thr hematite-binding motifs, which enable interaction with insoluble Fe(III) oxides (Lower et al., 2008). It shows weak homology (24% identity, 91% coverage) to decaheme c-type cytochrome MtrA of *Shewanella oneidensis*, which was proposed to localize on the outer membrane and form a wire connecting the periplasmic and extracellular environments (Richardson et al., 2012). Probably, this cytochrome is localized on the outer membrane of the SHF-111 bacterium and serves as a terminal Fe(III) reductase. Overall, the SHF-111 genome comprised six genes encoding multiheme c-type cytochromes containing hematite-binding motifs.

Two other gene clusters (3 and 4 in Figure 4) encode enzyme complexes resembling alternative complex III (ACIII), initially described in *Rhodothermus marinus*, where it functionally substitute cytochrome *bc1* complex III (Pereira et al., 2007). The gene clusters of ACIII usually contain six open reading frames (*actABCDEF*) encoding c-type cytochromes (ActA with five or six hemes and ActE with one heme), the catalytic subunit of complex iron-sulfur molybdoenzyme family (CISM) enzymes fused to iron-sulfur electron-transfer module (fused ActB or separate ActB1 and ActB2), and membrane-bound subunits (ActCDF) (Refojo et al., 2010). In *R. marinus* and other microorganisms where ACIII is a component of the aerobic respiratory chain, the *act* operon is clustered with cytochrome c oxidase genes, but this is not the case for ACIII, which transfer electrons to periplasmic acceptors (Yanyushin et al., 2005; Refojo et al., 2010). In the SHF-111 genome both *act*-like gene clusters are located distantly from the cytochrome c oxidase loci suggesting their irrelevance to oxygen respiration. Cluster 3 comprises two copies of *actABCD* genes and single genes *actE* and *actF* (Figure 4). Cluster 4 comprises genes for subunits ActABCD and ActE-like protein containing four rather than one heme. In addition, this locus contains two counter oriented operons encoding two subunits of the CISM family oxidoreductase, the 4Fe-4S ferredoxin iron-sulfur protein and the NrfD-like membrane protein forming quinol-oxidizing module, as well as multiheme c-type cytochrome instead of molybdopterin-binding catalytic subunits (Figure 4).

Another complex comprises iron-sulfur/membrane anchor module and multiheme c-type cytochrome (cluster 2 in Figure 4). Similar complexes found in the iron-reducing bacterium *Melioribacter roseus* were proposed to serve as quinol: electron acceptor reductases that donate electrons to periplasmic multihaem c-type cytochromes (Podosokorskaya et al., 2013). This electron transfer chain may terminate at the hypothetical Fe(III) reductase linked to the outer membrane.

### Resistance to heavy metals and arsenate

Several mechanisms of resistance to metal stress were identified in the SHF-111 genome. The genome contains two P-type ATPases of the TC 3.A.3 superfamily transporting copper, lead, cadmium, zinc, and mercury, a chromate ion transporter (TC 2.A.51), a Cd^2+^/Zn^2+^ efflux pump of the cation diffusion facilitator (CDF) Family (TC 2.A.4), CorA Mg^2+/^/Co^2+/^/Ni^2+^ transporter of the Metal Ion Transporter Family (TC 1.A.35), CorC exporter (TC 1.A.112), and MntP manganese efflux pump (TC 2.A.107). The arsenate resistance operon includes genes for transcriptional regulators (*arsD* and *arsR*), soluble arsenate reductase (*arsC*) which reduces arsenate to arsenite, arsenical pump-driving ATPase (*arsA*) and ACR3 family arsenite efflux transporter (*arsB*).

### Comparative genomics of the MBNT15 phylum

**FIGURE 5:**
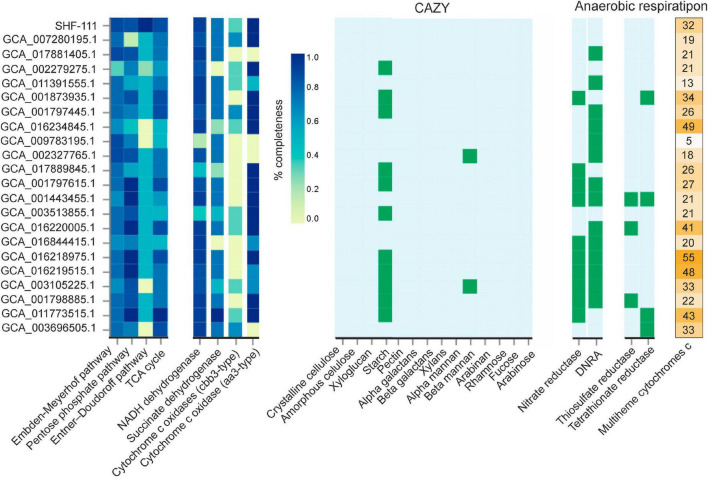
Comparative analysis of the main metabolic pathways along the candidate phylum MBNT15. The completeness of the metabolic pathways and the completion of the subunits of the respiratory complexes were assessed using DRAM. The presence/absence of carbohydrate-active enzymes and enzymes involved in dissimilatory reduction of nitrogen and sulfur compounds was also detected using DRAM. The rightmost column shows the number of multiheme cytochromes c present in the genomes. DNRA, ammonia-forming cytochrome c nitrite reductase.

At present, the candidate phylum MBNT15 comprises 22 species defined on the basis of the corresponding MAGs (Figure 2). In order to get insights into the metabolic diversity of MBNT15 division, we analyzed the presence of key genetic determinants and pathways across all of these MAGs (Figure 5). Although only the SHF-111 genome is 100% complete, nine other genomes are high quality assemblies (>90% completeness) and the remaining 12 are medium quality assemblies (65–90% completeness); therefore the absence of a certain trait should be considered with caution and can be explained by the incompleteness of the assembly.

All members of MBNT15 seem to have the Embden–Meyerhof glycolytic pathway and the TCA cycle. Pentose phosphate pathway was complete or near complete in all MAGs with the exception of PowLak16_MAG18, the closest relative of SHF-111, in which only 1 of 7 genes was found. However, this MAG was only 76.6% complete. The Entner–Doudoroff pathway was completely absent from the four MAGs, and they were all high quality assemblies so accidental absence of these genes is unlikely.

Like the SHF-111 genome, none of the MBNT15 bacteria were predicted to be able to utilize complex polysaccharides, including cellulose, xylan, pectin, mannan, and arabinan, or to grow on fucose, rhamnose, and arabinose. However, unlike SHF-111, almost half of the MBNT15 genomes contain glycoside hydrolases (e.g., GH13, GH15, GH57, and GH133) involved in the degradation of starch and similar polysaccharides.

Cytochome c oxidases were found in all MBNT15 genomes except for two MAGs of the genus g_UBA2219 and MAG Chersky.9_15, belonging to the same genus as the SHF-111 bacterium. Low affinity *aa3*-type oxidases were more widespread than high affinity *cbb3*-type enzymes: these two types of cytochome c oxidases were found in 17 and 11 genomes, respectively. Interestingly, the inventory of cytochome c oxidases can vary even within the same genus.

Most members of MBNT15 possess genes enabling anaerobic respiration with nitrogen compounds, but their set differs markedly among genomes (Figure 5). Genes for NarGHI-type nitrate reductase were found in nine genomes, one genome encodes NapAB-type nitrate reductase, and genes for ammonia-forming cytochrome c nitrite reductase (NrfAH) were present in 14 genomes. Seven genomes contain the complete denitrification pathway from nitrate to ammonia. The ability to use oxidized sulfur compounds for anaerobic respiration appeared to be rare among MBNT15 species. None of the genomes encodes dissimilatory sulfate reduction pathway. Thiosulfate and tetrathionate reductases were identified in 3 and 4 genomes, respectively.

Analysis of the MBNT15 genomes has shown that dissimilatory iron refection is likely the most common and ancestral respiratory pathway in this phylum. This is evidenced by the observation that 22 out of 23 genomes contain a large number of multiheme cytochromes c, from 13 to 55 per genome (Figure 5), and the number of hemes in individual proteins reaches 43 units (Supplementary Table 5). The only exception is MAG Nt197P3bin122 (GCA_009783195.1) encoding only five multiheme cytochromes c with up to seven hems; their predicted functions were not related to iron reduction. Unlike other MBNT15 genomes, this MAG was assembled from the termite gut metagenome, an anaerobic environment where high content of Fe(III) is not expected. Consistently, cytochrome *c* oxidases were also absent in this genome. Probably in the course of evolution and adaptation to a strictly anaerobic organic-rich environment, this species lost the genetic determinants enabling aerobic respiration and iron reduction, and switched to a fermentative anaerobic lifestyle.

### Description of new taxa

The genome of the SHF-111 bacterium meets the criteria, suggested for the description of new taxa of uncultivated prokaryotes (Konstantinidis et al., 2017), and we propose the following taxonomic names for the novel genus and species of SHF-111.

Description of the novel genus *Candidatus* Deferrimicrobium (De.fer.ri.mi.cro’bi.um. L. pref, *de*, from; L. n. *ferrum* iron; N.L. neut. n. *microbium* microbe; N.L. neut. n. Deferrimicrobium, microbe that reduces iron).

Description of the novel species *Candidatus* Deferrimicrobium borealis (bo.re.a’lis. L. fem. adj. *borealis* pertaining to the north, boreal).

Not cultivated. Inferred to be rod shaped, facultatively anaerobic organotroph that obtains energy by fermentation, aerobic respiration or dissimilatory iron reduction, and capable of using proteinaceous substrates, fatty acids, di- and tricarboxylates of the tricarboxylic acids cycle and some sugars as growth substrates. Represented by a near-complete genome (GenBank acc. no. JAMHFW000000000) obtained from the metagenome of eutrophic fen in the North European Russia.

Based on this, we propose the following names for the class, order, and family:

*Candidatus* Deferrimicrobia classis nov.

*Candidatus* Deferrimicrobiales ord. nov.

*Candidatus* Deferrimicrobiaceae fam. nov.

Assuming the results of phylogenetic analysis, and because of the appearance of a representative with a known complete genome sequence, the candidate phylum MBNT15 is proposed to be named as *Candidatus* Deferrimicrobiota phyl. nov., following recommendations suggested in Whitman et al. (2018). The phylum *Candidatus* Deferrimicrobiota is defined on a phylogenetic basis by comparative genome sequence analysis of *Candidatus* Deferrimicrobium borealis SHF-11 and multiple uncultured representatives, inclusive of the formerly identified candidate division MBNT15.

## Conclusion

This study provides the first insight into the biology of a member of the candidate phylum MBNT15 found in a boreal peatland and contributes to the understanding the phylogenetic and metabolic diversity of this lineage. According to the results of 16S rRNA gene profiling, members of the MBNT15 accounted for up to 2% of microbial communities in peat samples from eutrophic fens but were absent in nearly located raised bogs.

We assembled the MAG of a member of the MBNT15 from the peat metagenome and used genomic data to analyze the metabolic pathways of this bacterium, designated SHF-111. This bacterium was predicted to be rod shaped, it lacks flagellar machinery but twitching motility was encoded. Analysis of the SHF-111 genome revealed the presence of redundant glycolytic pathways, including the Embden–Meyerhof, Entner–Doudoroff and pentose phosphate pathways, as well as the complete TCA cycle. The SHF-111 bacterium was predicted to be specialized in the utilization of low molecular weight organic substrates, including peptides, amino acids, di- and tricarboxylates of the TCA cycle (citrate, succinate, fumarate, and malate), fatty acids, glycerol, some mono- and disaccharides. The SHF-111 genome encodes the electron transfer chain for aerobic respiration ending with cytochrome c oxidases of *aa3* and *cbb3* types and cytochrome *bd* oxidase. These enzymes are known to have different affinities for oxygen, and their presence can ensure the survival of the SHF-111 bacterium at various oxygen concentrations. The SHF-111 genome contains 32 genes encoding multiheme c-type cytochrome with up to 16 heme-binding motifs which probably enable its growth by dissimilatory iron reduction. Consistently, the relative abundance of MBNT15 in peatlands positively correlated with iron concentration. Apparently, in the wetland ecosystem, MBNT15 representatives play the role of scavengers, carrying out the complete mineralization of low molecular weight organic substances formed as a result of microbial degradation of complex polymeric substrates.

Comparative genome analysis of the MBNT15 phylum revealed that the vast majority of its members are capable of aerobic respiration, and some species are also capable of anaerobic respiration using oxidized sulfur and nitrogen compounds. Dissimilatory iron reduction, as evidenced by the abundance of multiheme c type cytochromes, is a hallmark of the MBNT15 phylum, found in 22 out of the 23 analyzed genomes. Based on phylogenetic and genomic analyses, the novel bacterium is proposed to be classified as *Candidatus* Deferrimicrobium borealis, within a candidate phylum Deferrimicrobiota.

## Data availability statement

The datasets presented in this study can be found in online repositories. The names of the repository/repositories and accession number(s) can be found below: https://www.ncbi.nlm.nih.gov/genbank/, SRX15240387 https://www.ncbi.nlm.nih.gov/genbank/, SRX15240388 https://www.ncbi.nlm.nih.gov/genbank/, JAMHFW000000000.

## Author contributions

NR: conceptualization, supervision, and funding acquisition. SB, AB, and AM: investigation. SD: resources. NR and AB: data curation. SB, AM, and SD: validation. SB and NR: writing–original draft preparation and writing–review and editing. All authors read and agreed to the published version of the manuscript and approved the submitted version.

## Footnotes

1
https://github.com/umerijaz/microbiomeSeq

2
https://github.com/najoshi/sickle

3
http://www.cbs.dtu.dk/services/TMHMM/
